# Physico-Chemical Characteristics and Lipid Oxidative Stability of Zebra (Equus Burchelli) Droëwors Made Using Different Levels of Sheep Fat

**DOI:** 10.3390/foods10102497

**Published:** 2021-10-18

**Authors:** Zikhona Mandela, Elodie Arnaud, Louwrens C. Hoffman

**Affiliations:** 1 Department of Livestock and Pasture Science, University of Fort Hare, Private Bag X 1314, Alice 5700, South Africa; zmandela@ufh.ac.za; 2 Department of Animal Sciences, Stellenbosch University, Private Bag X1 Matieland, Stellenbosch 7602, South Africa; louwrens.hoffman@uq.edu.au; 3 CIRAD, UMR QualiSud, Matieland, Stellenbosch 7602, South Africa; 4 Qualisud, Univ Montpellier, Avignon Université, CIRAD, Institut Agro, Université de La Réunion, F-34398 Montpellier, France; 5 Queensland Alliance for Agriculture and Food Innovation (QAAFI), The University of Queensland, Digital Agricultural Building, 8115, Office 110, Gatton 4343, Australia

**Keywords:** dry sausages, fat levels, nitrite free, game meat, wild meat, storage, proximate, water activity, TBARS

## Abstract

The physico-chemical properties (proximate, salt content, water activity (a_w_), pH) and lipid oxidation of droëwors (dried salted/spiced meat sausages) produced with zebra meat and different sheep fat levels (10, 15, and 20% by weight) measured at day 0 (before drying), day 2 (after drying at 30 °C and 40% relative humidity), and over a 90 day storage (day 17, 32, 47, 62, 77, and 92) under vacuum at 25 °C were investigated. The use of lower fat levels (10 and 15%) in the formulation resulted in higher weight loss during drying and droëwors with higher protein, ash, and salt content and lower a_w_ and pH compared to the droëwors made with 20% fat. The pH increased (*p* < 0.001) during storage for all the fat levels, while the moisture content and the a_w_ were stable as expected. TBARS values were the highest in droëwors made with 20% of fat after drying (day 2), but droëwors made with 10% of fat reached similar maximal values on day 17. Formulations containing 15% sheep fat displayed the lowest TBARS values after drying and along storage, and thus had the best characteristics in relation to oxidative stability.

## 1. Introduction

Droëwors is a traditional South African shelf-stable salted and dried meat sausage produced from a combination of minced meat (beef, ostrich, or game species), fat, spices, and vinegar [1,2]. The mixture is stuffed into natural casings before being dried for a few days [3]. Drying is the oldest method of meat preservation, affecting the product characteristics by reducing the moisture content and its water activity so that microbial growth is limited [4]. Droëwors is a product produced predominantly for the domestic market, yet the quality of the product varies widely, and it is difficult to formulate quality standards due to the lack of published scientific literature.

Animal fat is added into meat products to improve texture, taste, and flavour [5,6], and allows the constant removal of water during drying [7]. Nonetheless, concerns raised by consumers about processed meat products with high levels of saturated fat [8] has led to wide investigations on fat reduction in these products. Consumers demand healthier and high-quality food products, which require lower fat levels whilst flavour and juiciness are maintained. Reduction of fat intake is recommended because of the adverse effects for human health through excessive consumption of fat, such as obesity and coronary heart diseases [9,10].

The use of game meat, such as zebra, in the production of droëwors has not been studied. Zebra meat is considered to be low in fat and cholesterol and rich in protein [11], with a desirable fatty acids’ (unsaturated) profile [12]. Utilising game meat holds a potential in contributing to food security and economic stability in South Africa [13,14] and the rest of the world [15].

Beef fat (brisket) and sheep fat are commonly used in droëwors production; pork is hardly ever used due to increased rancidity, as showed by Mukumbo et al. [2]. Sheep fat, particularly that originating from fat-tailed indigenous breeds are saught after for the production of fresh “boerewors”, a South African fresh meat sausage. This is due to the enhanced flavour obtained from the fat of these sheep breeds. With the increase in game ranching, the meat from various wildlife species, including that from zebra meat, has become more readily available for consumption as fresh meat. These meats are also used in processed products [16,17,18,19,20]. Lipid oxidation is an important factor to consider in droëwors, as high fat products stored for long periods are more prone to lipid oxidation, especially in the case of meat products stored at ambient temperature such as droëwors. Lipid oxidation is affected by various factors during meat processing, such as grinding, salting, and seasoning [21]. The lipid prooxidant effect of salt might be amplified by the reduction in moisture and the resulting increase in salt concentration during the drying of droëwors. Moreover, nitrites which have an antioxidant effect, as they act as oxygen scavengers when oxidized to nitrates [22], are not added in droëwors unlike many dried pork products. Lipid oxidation can result in quality losses in meat and meat products by limiting the nutritional quality, affecting the flavour, colour, and odour, which can pose as a threat to human health [23,24]. However, there is no substantive data validating that zebra droëwors made with sheep fat is indeed a shelf-life stable product. The current study, therefore, aimed to examine the physico-chemical composition and lipid oxidative stability of South African droëwors made from different sheep fat levels and zebra meat during storage under vacuum.

## 2. Materials and Methods

### 2.1. Droëwors Production and Storage

Droëwors were made with zebra (Equus burchelli) meat and sheep fat at three different meat:fat ratios (90:10, 85:15, 80:20) at the meat processing facility of Stellenbosch University. Zebra meat chunks (50 kg) were derived from 10 animals shot outside Bredasdorp, Western Cape, South Africa, as part of a commercial culling operation. The meat was cut into approximately 3 × 3 × 3 cm cubes after being trimmed of all external fat and connective tissue, thoroughly mixed, and placed into a large container. No specific carcass cuts/muscles were used. The same procedure was followed for the sheep back and tail fat (22 kg in total) derived from 10 fat-tailed type sheep.

Out of the large container of zebra meat, a total of eight replicate batches per fat level were produced separately. Each replicate batch was divided into three smaller batches of 2 kg, to which the three different levels of fat (treatment) were also randomly drawn for inclusion into the droëwors, according to the specific replicate’s recipe. Each batch of zebra and fat chunks were mixed by hand with spices (Freddy Hirsch, Cape Town, South Africa) before being ground through a 4.5 mm plate. Spices consisted of fine salt (1.5 g salt/100 g meat/fat mixture), coarse coriander seed (20 g/100 g meat/fat mixture), coarse black peppercorns (1 g/100 g meat/fat mixture), fine cloves (0.5 g/100 g meat/fat mixture), fine nutmeg (0.5 g/100 g meat/fat mixture), and brown vinegar (5% (*w*/*v*) acidity) at 2 g/100 g meat/fat mixture. The ground mixture was stuffed into sheep casings (±20–22 mm diameter), left to rest overnight (~12 h) at ± 4 °C, and dried in a temperature and humidity-controlled chamber at 30 °C and 40% relative humidity (RH) for 48 h ([2]). The weight of each batch was recorded before and after drying to calculate the weight loss. The dried sausages (droëwors) were packed under vacuum and stored in a chamber at 25 °C for 90 days, to determine lipid oxidation stability.

### 2.2. Sampling and Preparation of Samples

A 50 g sample was taken from each batch of droëwors at day 0 (raw batter before stuffing into casings) and after drying (day 2) for proximate, salt, water activity, pH, and lipid oxidation analysis. A representative 35 g sample was taken from each batch during storage on days 17, 32, 47, 62, 77, and 92 and analysed for moisture, water activity, pH, and lipid oxidation. All samples were homogenised individually in a blender (Ampa Cutter CT 35 N, Golasecca, Italy) for approximately 1 min. Each individual sample was roughly divided into two subsamples, one for lipid oxidation and one for other analyses, respectively stored at −80 °C and −20 °C until analysis.

### 2.3. Chemical Analysis

For proximate analyses, the samples were analysed according to AOAC [25] for moisture (Method 934.01) and ash content (Method 942.05). Total fat content was determined using the chloroform/methanol (2:1) fat extraction method according to Lee et al. [26]. The defatted and dried (48 h, 60 °C) samples were analysed for nitrogen using a LECO Nitrogen/Protein analyser (FP-528, Leco Corporation, 3000 Lakeview Avenue, St Joseph, MI, USA) (AOAC [27] procedure 992.15). Protein was calculated by multiplying the percentage of nitrogen by 6.25.

For determination of salt content, a 0.3 g sample was weighed into a closed container and agitated under stirring for at least 2 h with 50 mL of 0.3 M nitric acid. The chloride concentration was determined using a 926 Chloride analyser (Sherwood Scientific, Cambridge, UK).

A calibrated pH meter was used with the aid of a penetration electrode and temperature probe to measure the pH of the samples. A total of 3 g of ground sample was added to 27 mL distilled water in duplicate, mixed using a magnetic stirrer for 30 min. Thereafter, pH was measured during continuous stirring using a Crison PH25 pH metre (Crison Instruments, Barcelona, Spain), calibrated with pH 4 and pH 7 standard solutions at 25 ± 1 °C.

Water activity (a_w_) was measured in duplicate using an Aqua Lab Dew Point Water Activity Meter 4TE (Decagon Devices, Inc., Washington, DC, USA) at 25 °C.

Lipid oxidation was analysed by measuring the thiobarbituric acid reactive substances (TBARS) using a modified acid-precipitation technique, described by Mukumbo et al. [2]. The absorbance was measured at 530 nm in a Cecil CE2021 2000 Series spectrophotometer (Lasec SA (Pty) Ltd., Pretoria, South Africa). The TBARS values are expressed as mg malondialdehyde (MDA) equivalent (eq) per kg product.

All analyses were performed in duplicate.

### 2.4. Statistical Analysis

To test the effects of fat level and days on the various measurements, mixed model repeated measures ANOVAs were used. For the mixed model, fat level and days were treated as fixed effects, and the batches to which the treatments were applied as a random effect. Fisher LSD post hoc test was used to further analyse significant differences when the main effects/interaction effects were significant. Statistical analyses were conducted using the VEPAC module of Statistica 13. A 5% significance level was used as a guideline for determining significant differences.

## 3. Results and Discussion

### 3.1. Physico-Chemical Characteristics

The statistical significance (*p*-values) levels of the main effects and their interaction on the various physico-chemical parameters are indicated in Table 1. Interactions were noted for most of the measured attributes, excluding the fat and protein content. Day effect was significant on all the attributes (*p* ≤ 0.001). The fat level had an effect (*p* ≤ 0. 001) on ash, a_w_, and (*p* ≤ 0.05) protein and salt content.

Table 2 shows the physico-chemical characteristics of zebra droëwors made at the different meat:fat ratios (90:10, 85:15, 80:20) before (raw batter) and after drying (day 2). The raw batter made with 20% fat showed a lower moisture and protein content, and a higher fat content compared to the 90:10 and 85:15 treatments. As expected, significant differences were found before and after drying for all the treatments for moisture, protein, fat, ash, salt, a_w_, as well as for pH because of the weight/moisture loss during drying. The weight loss during drying decreased when fat level increased. This might be due to both the lower moisture content in the raw batter and the higher fat content, as fat may limit moisture transfers during drying. Finally, the moisture content of the droëwors differs only in the 85:15 and the 80:20 treatments (27.91 and 30.45 g/100 g, respectively). Higher weight loss during drying when fat content is decreased in the raw batter at levels close to this study has also been reported for other types of rapidly dried sausages [18] and short ripened fermented sausages [28]. Proximate composition of the droëwors was in the range of values reported on commercial beef, game, and ostrich droëwors [2]. Mean values for fat content ranged between 19.71 and 24.54% after drying, and was higher for the meat fat ratio 80:20 because of the higher fat content in the raw batter and despite the lower weight loss during drying. Protein mean values across treatments were 40.03–46.22 g/100 g in droëwors after drying (57.56–64.96 g/100 g dry matter).

Results for the effect of storage time (day 2–92) on moisture and a_w_ for zebra droëwors with different fat levels are presented in Table S1 (Supplementary Materials). The a_w_ and moisture content are one of the determinants of prolonged shelf-life in meat products [29]. As expected, there was no evolution regarding moisture content (26.60–30.45 g/100 g) and a_w_ (0.878–0.907) during storage, due to the vacuum packaging. Mukumbo et al. [2] reported a continuous decrease in moisture and a_w_ during storage of droëwors in open packaging, i.e., paper bags at room temperature from 23.6 to 7.1% and 0.81 to 0.47, respectively.

**Figure 1:**
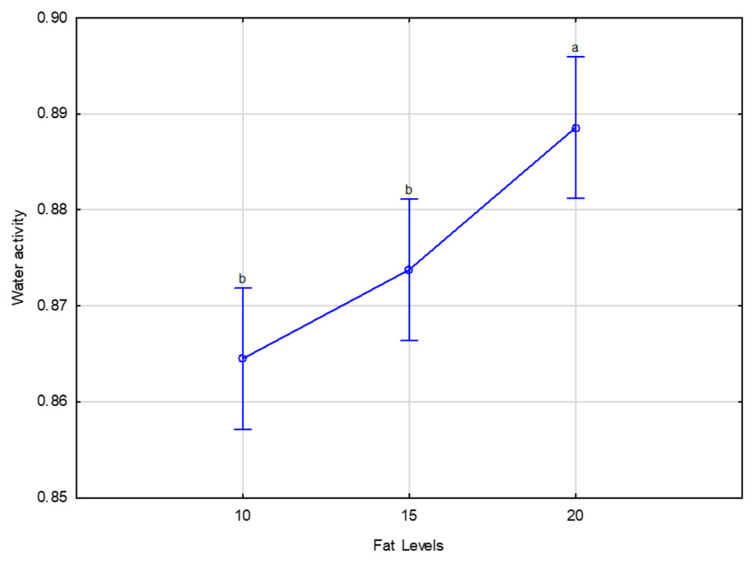
Water activity of zebra droëwors made with different sheep fat levels (means of day 2–day 92). ^ab^ Means with different superscripts differ (*p* ≤ 0.001).

There were no significant differences with increasing levels of fat amongst treatments for moisture during storage, while the a_w_ increased (*p* < 0.001) with fat level (Figure 1), although all the values obtained are in accordance with the requirements of a_w_ < 0.91 for meat to be storable at ambient temperature. Leistner and Rodel [30] classified meat products based on their pH and a_w_ values into “perishable” and “shelf-stable”. The “shelf-stable” meat products have an a_w_ < 0.91 or a pH < 5.2 and a_w_ < 0.95; no refrigeration is needed for these products, and their shelf-life stability is usually not restricted by bacterial growth but by physical or chemical spoilage, more specifically product discolouration and rancidity [31]. The a_w_ for the higher fat treatment (20% fat: 0.885 in average for days 2–92) indicates a greater possibility for microbial growth in products with high levels of fat compared with low fat levels (in this case, 10% fat: 0.864 and 15% fat: 0.874). In fact, if most of the bacteria do not grow when a_w_ is decreased to 0.91, Staphylococcus aureus can grow until a_w_ is decreased at 0.85 and 0.6–0.70 for yeasts and mould [4,32].

The a_w_ of droëwors is higher than values reported on commercial droëwors by Mukumbo et al. [2] (0.67–0.86), which is probably due to their lower salt content. The a_w_ was rather similar to that of dry fermented sausages (0 85–0.91; [33]). However, in dry fermented sausages, pH is another hurdle to microbial contamination.

**Figure 2:**
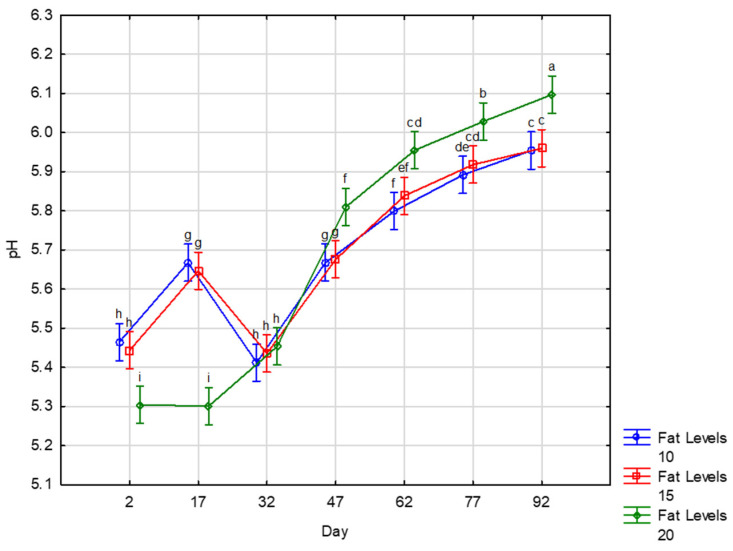
pH of zebra droëwors made with different sheep fat levels during storage. ^a–h^ Means with different superscripts differ (*p* ≤ 0.001).

The pH showed significant values for storage days’ effect and the interaction of fat level and storage days (Figure 2). The pH of droëwors after drying ranged from 5.30–5.46 (Table 2), and increased significantly during storage. Mukumbo et al. [2] reported values ranging between 4.9 to 5.7 on commercial products. Up to day 47 (45 days of storage), the pH was still in this range. We did not measure pH as low as 4.9, as was noted in the study of Mukumbo et al. [2], their lower readings might be due to the use of higher volumes of vinegar compared to our study (2% of vinegar 5% *w/v* acidity). In fact, the same formulation in biltong and droëwors is used by processors, as droëwors are currently processed from off cuts of the meat used in the production of biltong. Up to 5% of vinegar (5% acidity) has been reported in the processing of biltong and its addition resulted in the pH decreasing to values lower than 5 [34].

The increase in the pH of the droëwors during storage is in accordance with the findings of Mukumbo et al. [35]. These authors reported an increase from 5.3 to 6.0 within the first 10 days of storage of pork droëwors stored 2 months unpacked in ambient conditions. A smaller increase in pH (0.3 unit) is often noticed at the end of ripening of mould ripened fermented sausages, after its reduction to values close to 5.0 throughout the first days of fermentation. This is due to the utilisation of lactate and acetate and the production of ammonia from amino acid breakdown [36]. A pH higher than 6 was recorded in some dry fermented sausages, such as traditional pork sausages [37], dry fermented sausages made from deer [38], and some game meat, such as the meat from black wildebeest which is known to be easily stressed [39]. The pH of droëwors made with 20% fat, which was lower at the end of drying compared to the two other treatments, exhibited a higher increase in pH and showed higher values from day 47 until the end of storage. This might be related with its higher a_w_, as microorganisms are involved in proteolytic phenomena, and its lower salt content as salt acts as an inhibitor of muscle proteolytic activity [7].

### 3.2. TBARS

**Figure 3:**
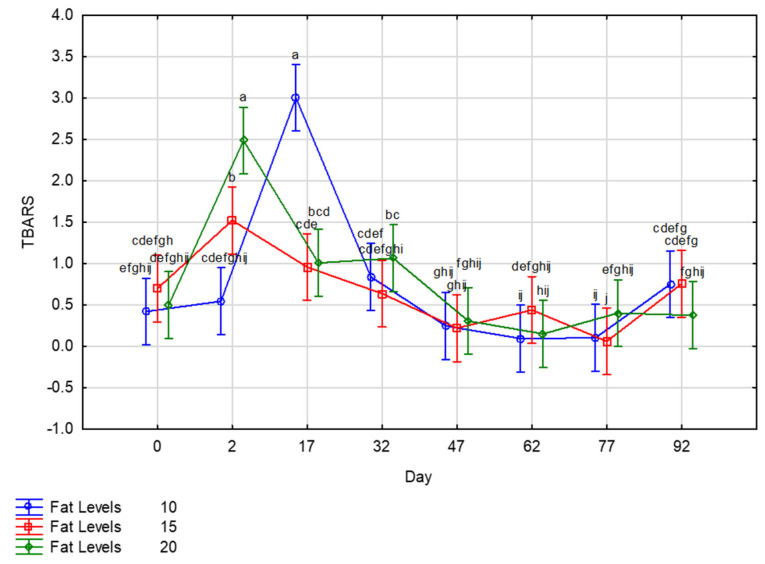
TBARS of zebra droëwors made with different sheep fat levels before drying (day 0), after drying (day 2), and during storage at 25 °C, under vacuum (day 17–92). TBARS: MDA mg eq/kg (eq = equivalent). ^a–j^ Means with different superscripts differ (*p* ≤ 0.05).

The results of lipid oxidation for zebra droëwors (before, after drying, and during storage at 25 °C, under vacuum) are depicted in Figure 3. Differences (*p* < 0.001) are seen in the droëwors before and after drying, between treatments, and throughout storage. Before drying (day 0), TBARS were similar for all sheep fat levels. During drying and successive storage, TBARS increased then decreased, as shown in pork droëwors [2,35] and other dry meat products [40,41,42]. This phenomenon in measured TBARS is attributed to the fact that MDA is an intermediate product in lipid oxidation, and can react with proteins [43]. After drying (day 2), TBARS values were highest in droëwors made with 20% of fat and TBARS decreased with fat level, but droëwors made with 10% of fat reached similar maximum values on day 17. The maximum value measured for the 15% fat treatment (1.5 mg eq MDA/kg) was lowest than the maximum value measured for the two other treatments. However, the maximal value might have been reached between the samplings of day 2 and 17. The 80:20 and 90:10 had similar maximum TBARS values. Despite its lower fat content, the 90:10 treatment had higher salt content and higher pH, and it has been showed that lipid oxidation increased as the pH of meat decreased to from 7 to 5.9 [44]. 

Different types of meat and fat have been studied in droëwors production and TBARS values have been followed at different stages. Jones et al. [45] reported lower TBARS (0.7–0.9 mg MDA eq/kg at the end of 2 days drying) in droëwors made from other game species (blesbok, springbok, and fallow deer) and sheep fat (30%). Mukumbo et al. [2] produced and stored droëwors from beef and pork, together with beef and pork fat and reported higher TBARS values for pork droëwors after drying and during storage at 25 °C without packaging (1.8–3.8 mg MDA eq/kg dry matter), as compared with beef droëwors (<1 mg MDA eq/kg dry matter). Differences in TBARS values were explained by the saturated fat profile in beef fat compared to that from pork with the latter fat source being more susceptible to rancidity. TBARS of pork fermented dry sausages reached lower values (often less than 1, sometimes up to 2 mg MDA eq/kg [46]) as did sheep fermented sausages [47,48], however it should be remembered that droëwors are processed without nitrites/nitrates and at a higher temperature. The increase in TBARS with fat level in fermented dry sausages have already been reported [46,49].

According to Campo et al. [50], rancidity is detectable when TBARS values are higher than or equal to 2 mg MDA eq/kg of fresh meat, although Utrilla et al. [51] reported that values up to 5 mg MDA eq/kg are acceptable whilst 10 mg MDA eq/kg is unacceptable. This is in accordance with the study of Hoffman et al. [52], in which the TBARS of droëwors over a drying period of 15 days produced with ostrich meat and 25% pork fat reached 7–11 mg MDA eq/kg, while no rancidity was detected by the panel. In this regard, droëwors for all treatment levels gave acceptable results in terms of lipid oxidation towards the end of storage (day 32–92).

## 4. Conclusions

A higher weight loss was obtained for the 90:10 and 85:15 treatment compared to the 80:20 treatment, and their fat content were lower as a result of their lower fat content in the raw batter and despite the higher moisture loss during drying. pH ranged between 5.3–5.46 after drying, despite the addition of vinegar and a higher increase in pH during storage was measured for the droëwors made with 20% of fat as well as higher values from day 47 until the end of storage. This might be due to higher proteolytic activity related with its higher a_w_ and lower salt content. These values were however still in the range for shelf-stable products (>0.91), but a recommendation could be to reduce a_w_ by increasing salt content in the formulation at 2% and/or to add more vinegar, as additional salt might have health implications.

An interesting trend was observed for the lipid oxidation of droëwors across treatments. TBARS values increased significantly after drying or the early days of storage at 25 °C under vacuum (up to 3 mg MDA eq/kg), but decreased and remained at a minimum during storage whatever the fat level. Furthermore, the highest fat level (20%) led to a higher lipid oxidation after drying, but similar maximal values were reached later with the droëwors made with 10% of fat. This seems to be higher than TBARS values recorded in droëwors made with other meat/fat (except those made with pork meat and fat) or fermented sausages. However, the impact of such high values on the detection of rancidity by a sensory panel or consumers should be studied. With the growing recognition of quality and sensory properties of meat products, consumer and sensory evaluation of zebra droëwors made with different levels of sheep fat should be evaluated. Furthermore, future research could be conducted on the fatty acid profile of this product, as fat reduction or utilisation of a healthier fatty acid profile could be an advantage in the production of droëwors, making it a healthy ready-to-eat meat product and thus playing a role in the formulation and shelf-life stability of droëwors.

## Figures and Tables

**Table 1:** Level of statistical significance (*p*-values) for the main effects of sheep fat level, day, and their interactions on physico-chemical characteristics and TBARS of zebra droëwors.

Attributes	Fat Level Effect	Day Effect	Fat Level × Day
moisture	0.694	≤0.001	≤0.001
protein	0.030	≤0.001	0.48
fat	≤0.001	≤0.001	0.100
ash	≤0.001	≤0.001	0.030
salt	≤0.010	≤0.001	≤0.001
water activity	≤0.001	≤0.001	≤0.010
pH	0.66	≤0.001	≤0.001
TBARS	0.55	≤0.001	≤0.001

**Table 2:** Weight loss during drying and physico-chemical properties (mean and standard errors, *n* = 8) of raw batter and zebra droëwors made with different sheep fat levels.

	Raw Batter (before Drying)			Droëwors after Drying ^1^		
Meat Fat Ratio	90:10	85:15	80:20	90:10	85:15	80:20
weight loss	-	-	-	55.84 ^a^ ± 0.44	52.34 ^b^ ± 0.39	49.63 ^c^ ± 0.60
moisture	69.70 ^a^ ± 0.23	68.46 ^a^ ± 0.64	64.82 ^b^ ± 0.26	29.56 ^cd^ ± 0.88	27.91 ^d^ ± 0.71	30.45 ^c^ ± 1.11
protein	20.98 ^d^ ± 0.49	19.81 ^d^ ± 0.66	16.61 ^c^ ± 0.93	45.76 ^a^ ± 0.95	46.22 ^a^ ± 0.84	40.03 ^b^ ± 1.58
fat	6.97 ^e^ ± 0.70	9.30 ^d^ ± 0.73	13.67 ^c^ ± 1.17	19.71 ^b^ ± 0.61	19.99 ^b^ ± 0.81	24.54 ^a^ ± 1.13
ash	2.16 ^c^ ± 0.27	1.85 ^c^ ± 0.05	1.91 ^c^ ± 0.12	5.48 ^a^ ± 0.29	4.57 ^a^ ± 0.06	4.10 ^b^ ± 0.22
salt	0.94 ^c^ ± 0.03	0.93 ^c^ ± 0.01	0.94 ^c^ ± 0.01	2.02 ^a^ ± 0.07	1.91 ^a^ ± 0.04	1.69 ^b^ ± 0.04
water activity	0.986 ^a^ ± 0.00	0.985 ^a^ ± 0.00	0.987 ^a^ ± 0.00	0.878 ^c^ ± 0.01	0.881 ^c^ ± 0.01	0.907 ^b^ ± 0.01
pH	5.95 ^a^ ± 0.09	5.86 ^a^ ± 0.00	5.85 ^a^ ± 0.01	5.46 ^b^ ± 0.01	5.44 ^b^ ± 0.02	5.30 ^c^ ± 0.01

Weight loss, moisture, protein, fat, ash, salt contents: g/100 g. ^a–e^ Means in the same row with different superscripts differ (*p* ≤ 0.05). ^1^ Drying conditions: 30 °C, 40% RH, 48 h.

## Data Availability

Data from this study can be obtained from the corresponding author.

## Supplementary Materials

The following are available online at https://www.mdpi.com/article/10.3390/foods10102497/s1, Table S1. Moisture and water activity (mean and standard errors, *n* = 8) of zebra droëwors made with different sheep fat levels after drying (day 2) and during storage (day 17–92).
