# Supplementary material for: Physico-Chemical Characteristics and Lipid Oxidative Stability of Zebra (Equus Burchelli) Droëwors Made Using Different Levels of Sheep Fat
Source: Foods. 2021 Oct 18;10(10):2497. doi: 10.3390/foods10102497 (PMC8536107; doi:10.3390/foods10102497)
Supplement: Supplementary file 1 [file foods-10-02497-s001.zip › foods-1396647-supplementary.pdf]

Table S1. Moisture and water activity (mean and standard errors, n=8) of zebra droëwors made with different sheep fat levels after drying (day 2) and during storage (day 17-92).

Day	After drying	Storage					
	2	17	32	47	62	77	92
**Meat fat ratio**							
				Moisture			
90:10	29.56[a] ± 0.88	28.98[a] ± 0.55	29.45[a] ± 0.71	28.94[a] ± 1.17	30.25[a] ± 0.73	29.33[a] ± 0.74	28.10[a] ± 0.61
85:15	27.91[a] ± 0.71	27.27[a] ± 1.17	29.28[a] ± 0.77	29.37[a] ± 0.57	26.60[a] ± 0.77	29.14[a] ± 0.60	29.60[a] ± 0.57
80:20t	30.47[a] ± 1.11	28.64[a] ± 1.04	28.15[a] ± 0.75	28.61[a] ± 0.92	29.79[a] ± 1.01	29.51[a] ± 0.73	30.21[a] ± 0.63
				Water activity			
90:10	0.878[a] ± 0.01	0.865[a] ± 0.01	0.865[a] ± 0.00	0.863[a] ± 0.01	0.866[a] ± 0.00	0.860[a] ± 0.00	0.854[a] ± 0.00
85:15	0.881[a] ± 0.01	0.873[a] ± 0.01	0.875[a] ± 0.01	0.874[a] ± 0.00	0.875[a] ± 0.00	0.872[a] ± 0.00	0.868[a] ± 0.00
80:20	0.907[a] ± 0.01	0.894[a] ± 0.01	0.882[a] ± 0.00	0.882[a] ± 0.00	0.888[a] ± 0.00	0.866[a] ± 0.00	0.882[a] ± 0.00

Moisture: g/100g

[a] Means in the same row with different superscripts differ (P ≤ 0.05)
